# Which Body Density Equations Calculate Body Fat Percentage Better in Olympic Wrestlers?—Comparison Study with Air Displacement Plethysmography

**DOI:** 10.3390/life11070707

**Published:** 2021-07-17

**Authors:** Aslı Devrim-Lanpir, Ebru Arslanoğlu Badem, Hatice Işık, Aslıhan Nefes Çakar, Banu Kabak, Bihter Akınoğlu, Tuğba Kocahan, Adnan Hasanoğlu, Thomas Rosemann, Beat Knechtle

**Affiliations:** 1 Department of Nutrition and Dietetics, Istanbul Medeniyet University, Istanbul 34862, Turkey; asli.devrim@medeniyet.edu.tr; 2 Department of Health Services, Sports General Directorship, Center of Athlete Training and Health Research, The Ministry of Youth and Sports, Ankara 06820, Turkey; ebru_arslanoglu@hotmail.com (E.A.B.); aslihannefes@gmail.com (A.N.Ç.); banu.kabak@gsb.gov.tr (B.K.); rgkardelen@yahoo.com (B.A.); kocahantu@gmail.com (T.K.); ahasanoglu@gmail.com (A.H.); 3 Department of Statistics, Hacettepe University, Ankara 06100, Turkey; haticeyeniay86@gmail.com; 4 Department of Physiotherapy and Rehabilitation, Faculty of Health Sciences, Ankara Yıldırım Beyazıt University, Ankara 06830, Turkey; 5 Department of Sports Medicine, Gulhane Faculty of Medicine, University of Health Sciences, Ankara 06010, Turkey; 6 Institute of Primary Care, University Hospital Zurich, 8091 Zurich, Switzerland; thomas.rosemann@usz.ch

**Keywords:** BOD POD, anthropometry, body composition, fat mass, wrestling, skinfolds

## Abstract

Although skinfold-derived equations seem to be practical for field application in estimating body fat percentage (BF%) and minimum body mass in Olympic wrestlers, prediction equations applied first need to be cross-validated in Olympic wrestlers to define the best prediction equation. This study aimed to evaluate the most accurate field method to predict BF% in Olympic wrestlers compared to BF% estimated by air displacement plethysmography (ADP). Sixty-one male (body mass 72.4 ± 13.5 kg; height 170.3 ± 7.0 cm; body mass index (BMI) 24.9 ± 3.5 kg.m^−2^; BF% 8.5 ± 4.9%) and twenty-five female wrestlers (body mass 60.3 ± 9.9 kg; height 161.3 ± 7.1 cm; BMI 23.1 ± 2.5 kg.m^−2^; BF% 18.7 ± 4.7%) undertook body composition assessments including ADP and nine-site skinfold measurements. Correlations, bias, limits of agreement, and standardized differences between alterations in BF% measured by ADP and other prediction equations were evaluated to validate measures, and multiple regression analyses to develop an Olympic wrestlers-specific prediction formula. The Stewart and Hannan equation for male wrestlers and the Durnin and Womersley equation for female wrestlers provided the most accurate BF% compared to the measured BF% by ADP, with the lowest bias and presented no significant differences between the measured and predicted BF%. A new prediction equation was developed using only abdominal skinfold and sex as variables, predicting 83.2% of the variance. The findings suggest the use of the new wrestler-specific prediction equation proposed in the study as a valid and accurate alternative to ADP to quantify BF% among Olympic wrestlers.

## 1. Introduction

Wrestling has been one of the oldest and most established parts of the Olympic games since ancient competitions [1]. Wrestling competitions are mainly monitored depending on the body mass class in order to ensure the revealing of physiological and psychological skills and to prevent physical characteristic advantages among wrestlers [2]. While this system seems fair to all wrestlers, wrestlers tend to apply a common strategy about body mass to gain a higher advantage from physical traits that include training with high body mass but rapid body mass loss just before a competition [3,4]. However, the fast body mass loss technique including fluid deprivation, sauna, fasting and dehydration may cause many undesirable consequences, including death [3,5].

The body mass management of wrestlers has been followed by the National Collegiate Athletic Association (NCAA) with certain standards since the death of three college wrestlers in 1997 [6]. The NCAA body mass management program requires to measure body composition and calculate body fat to monitor the lowest allowable body mass at five body fat percentage (BF%) in wrestlers [7]. Therefore, close monitoring of wrestlers’ body composition from the beginning of the training period to competition provides crucial benefits in eliminating fast body mass-loss strategies and maintaining ideal body composition throughout all processes [2,8]. In addition, a short-limbed physique and low BF% is commonly considered as the most advantageous biomechanical and physical characteristics for the wrestlers [9]. Furthermore, body fat percentage has been also widely used in field-based practice to predict minimum body mass in wrestlers [10,11]. Therefore, BF% is considered to be crucial, especially by coaches and wrestlers throughout the entire season.

Hydrostatic weighting (HW), dual-energy X-ray absorptiometry (DXA), and air displacement plethysmography (ADP) are well-proven techniques providing the most accurate determination of BF% in athletic populations [12]. However; since these devices require high financial costs and advanced technical settings, not all sports professionals can be able to apply them to monitor the athletes’ body composition [11,12]. Therefore, a number of skinfold equations have been implemented to estimate body fat in athletic populations [13,14,15,16,17,18]. While the skinfold equations previously developed by Durnin and Womersley [19] and Jackson and Pollock [20,21] are widely applied to estimate BF%, many equations were later developed on athletic populations. The NCAA approved the use of the Lohman skinfold equation developed on wrestlers as a field method to determine BF% [22]. In addition, Thorland et al. [15] verified a new equation for predicting BF% in wrestlers.

It should be noted, however, that not all skinfold equations give accurate results for estimating body fat in all athletic populations [23,24,25]. A skinfold equation can yield different results after cross validation in athletes who do even the same sport but have different ages, sexes and characteristics [12]. Therefore, verifying the skinfold equations in the athletic group before applying them will provide more accurate results to estimate BF% [26]. Given that body composition varies according to age, sex, and training experience [12], and closely monitoring changes in body composition is a certain need due to training and/or dietary interventions, there is a need for population-specific data for Olympic wrestlers.

To our knowledge, no studies have investigated the validity of skinfold equations in Olympic wrestlers. The hypothesis of the present study was that the accuracy of the skinfold equations applied to estimate BF% in athletes may vary according to gender and age groups. Therefore, the study aimed to validate the use of 12 common skinfold equations in Olympic wrestlers by gender and age group to evaluate which equation correlates best BF% measurement by BOD POD, to cross-validate two equations (Lohman [22] and Thorland et al. [15] ) previously developed for wrestlers, and to develop a skinfold prediction equation to accurately estimate BF% using the field anthropometric variables in Olympic wrestlers.

## 2. Material and Methods

### 2.1. Participants

Sixty-one male and 25 female Olympic wrestlers (Greco-Roman = 22, freestyle = 64) participated in the study. All participants were recruited from the National Olympic wrestlers at the Center of Athlete Training and Health Research. All participants and their parents or legal representatives were fully informed about the study purpose, procedures, and potential risks of the study before participation, and written consent was obtained prior to starting the study. Inclusion criteria for this study were: (a) a National Olympic wrestler for at least 1 year; (b) in a good health state; (c) no chronic or systemic health problems; (d) not pregnant; (e) no implanted medical devices. In addition, all female participants were tested during the mid-follicular phase (7–9 days of the menstrual cycle) to eliminate the effects of different phases on body composition and standardize the menstrual phase [27]. Menstrual cycle was calculated starting the first day of their period. Wrestlers who have any current or chronic health problems, claustrophobic, pregnant or any implemented medical devices were excluded from the study.

### 2.2. Anthropometric Measurements

Each participant underwent all measurements the same day, at 6–8 in the morning, after a 12-h fast. All measurements were obtained in a euhydrated state. Hydration status was determined by measuring urine specific gravity (Usg) with a semi-automatic urine analyzer (The Mission^®^ U500 Urine Analyzer, San Diego, CA, USA). Usg of 1.020 g/mL or less was considered to be sufficiently hydrated.

Body height was measured with a wall-mounted stadiometer to the nearest 0.1 cm. Body mass were measured using a multi-frequency body electric impedance (MF-BIA) analyzer (MC-980, 1000 kHz, 0.1 accuracy, Tanita, Tokyo, Japan). To get more accurate results from the measurements, all participants were asked to apply the following multi-frequency body electric impedance analysis (MF-BIA) guidelines: (a) consume no caffeine (at least 4 h), alcohol (at least 2 h), and cigarettes (at least 2 h); (b) avoid high-intensity exercise at least 24 h before the measurements.

Nine skinfold thicknesses (triceps, biceps, subscapular, iliac crest, chest, midaxillary, abdominal, anterior thigh and medial calf) were measured using a Holtain caliper (Holtain, Crymych, UK) in accordance with the International Society for the Advancement of Kinanthropometry (ISAK) protocol [28]. The physiotherapist had previous 25 years of experience measuring skinfold thicknesses in Olympic athletes including wrestlers and was familiar with the ISAK protocol. Each thickness was measured two times on the right side of the body, with the median value recorded [29]. A third measurement was applied in case of any technical error of measurement was exceeded (>5% for skinfold thicknesses).

Body fat percentage was estimated using skinfold equations, including equations developed for the athletic population and, in particular, wrestlers. Twelve skinfold equations used to predict BF%. The Brozek equation was used to estimate BF% in the equation where body density was calculated from a skinfold equation [30].

### 2.3. Criterion Method

The data obtained from BOD POD^®^ (Life Measurement Inc., Concord, CA, USA) measuring body volume using air displacement plethysmography were evaluated as the criterion method. The BOD POD was calibrated prior to each measurement according to manufacturer’s instructions using a 50-L calibration cylinder. The calibration of the BOD POD body mass scale was performed weekly. All measurements were performed under controlled-air temperature (23 °C) and humidity (at least 20%). All participants were asked to wear minimal clothes (a tight-fitting swimsuit and cap) for the test. Participants first sat comfortably inside the BOD POD and continued breathing normally while body volume was measured. Two 50-s tests were performed while the participants were in the BOD POD. The estimated mean air volume in the chest and lungs during respiration was measured by the BOD POD using the standard pulmonary plethysmographic technique. At the end of the measurement process, the BOD POD software system automatically calculated percentage body fat from body density using the Brozek equation [30].

### 2.4. Statistical Analysis

Normality tests were verified using the Shapiro–Wilk test. The Hedges’g statistic was calculated to determine the effect size of standardized differences in BF%, as it has better small sample properties than Cohen’s *d*. Hopkin’s scale for determining the magnitude of the effect size was used where 0–0.2 = trivial, 0.2–0.6 = small, 0.6–1.2 = moderate, 1.2–2.0 = large, >2.0 = very large [31]. The Bland-Altman method was used to identify the 95% limits of agreement of the BF% for the BOD POD and prediction equations. Linear regression was utilized to evaluate proportional bias between BOD POD and the prediction equations using the procedures described by Bland and Altman [32]. The variance inflation factor (VIF = 1/(1−R^2^)) and the condition index were used to test the collinearity. All assumption was evaluated for multiple regression model. Statistical analyzes were performed using IBM SPSS Statistics, version 23.0 (IBM Corp., Armonk, New York, NY, USA) and R Studio using the “blandr” package. The statistical significance level was accepted as *p* < 0.05.

## 3. Results

The physical characteristics of Olympic wrestlers, the descriptive values of BF% measured by BOD POD^®^ and estimated by skinfold-based equations are presented in Table 1. For skinfold measurements, the technical errors of the measurements were ≤1.38% and the coefficients of variation were ≤0.88 (Table 2).

**Figure 1:**
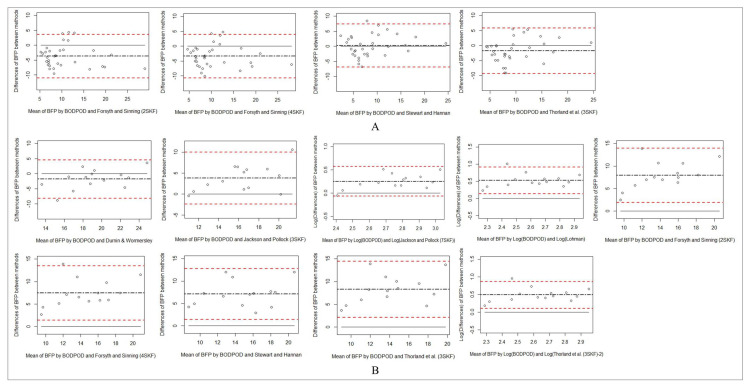
Bland–Altman plots for BODPOD and the prediction equations for wrestlers aged below 18. The dash-dotted line represents the mean difference (BIAS) between methods. The upper and lower dashed lines represent the 95% limits of agreement. The solid line represents the zero line. (**A**) Represents Bland-Altman plots of male wrestlers. (**B**) Shows Bland-Altman plots of female wrestlers.

**Figure 2:**
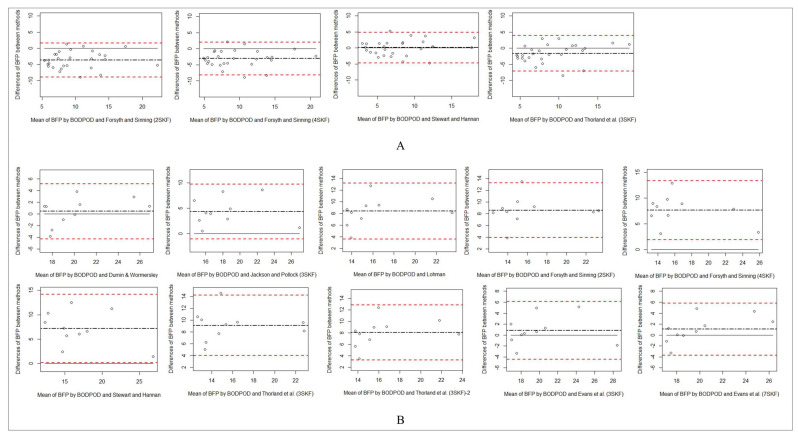
Bland–Altman plots for BODPOD and the prediction equations for wrestlers aged above 18. Bland–Altman plots for BODPOD and the methods for the subjects. The dash-dotted line represents the mean difference (BIAS) between methods. The upper and lower dashed lines represent the 95% limits of agreement. The solid line represents the zero line. (**A**) Represents Bland-Altman plots of male wrestlers. (**B**) Shows Bland-Altman plots of female wrestlers.

Table 3 represents correlations, bias, limits of agreement, and standardized differences between alterations in body fat percentage measured by BOD POD and other practical estimates in Olympic wrestlers. Bland-Altman plots are presented in Figure 1 and Figure 2 for inter-method agreement between the BF% data derived from the skinfold equations showing no bias and measured with the BOD POD^®^. The Stewart & Hannan equation showed no significant or standardized differences versus BOD POD and was positively correlated with BF% measured by BOD POD in male wrestlers. No differences and proportional bias detected between % body fat measured by BOD POD and the Durnin& Womersley equation in female wrestlers.

Correlations between % body fat measured by BODPOD and skinfold measurements in Olympic wrestlers are shown in Table 4. All skinfolds and sum of skinfolds revealed significant low to moderate positive correlations with BF% derived by BOD POD (ranging from 0.258 to 0.684; all *p* < 0.05), except for biceps.

Models developed to estimate BF% in Olympic wrestlers using linear regression analysis are shown in Table 5. Equations involving sex only or sex and abdominal skinfold revealed an adjusted R^2^ value of 0.635 and 0.827, respectively (*p* < 0.001). The new prediction equation that can be applied to estimate BF is (BF% = 0.30 + (0.72 × abdominal) + (11.43 × sex).

## 4. Discussion

This study examined twelve skinfold-derived equations against the BOD POD to determine the most accurate field method that can be used to estimate BF% among Olympic wrestlers. The major findings of the study were as follows: (1) the Stewart and Hannan equation for male Olympic wrestlers and the Durnin and Womersley equation for female Olympic wrestlers were the most accurate, least biased, and positively correlated with the BF% measured by BOD POD; (2) an equation for Olympic wrestlers explained 83.2% of the variance in BOD POD BF% using sex and abdominal skinfold measurement.

This study tested nine skinfold-derived equations developed for athletes—all athletes, male athletes, adolescent athletes, college football players, adult wrestlers, and high school wrestlers—as well as three equations developed on sedentary individuals. The Stewart and Hannan equation [14], developed on 82 well-experienced male athletes, including 63 local clubs and 19 internationals, provided the most accurate prediction for male wrestlers with the closest BF% to BOD POD, the lowest proportional bias, and no significant differences between the measured and predicted BF%. For female wrestlers under 18 years of age, only Durnin-Womersley [19] showed no significant difference between measured and estimated BF percentage, although the other equations fit well with BF% derived from BOD POD. Although the Durnin and Womersley [19] and the Evans equations (3 SKF and 7 SKF) [18] showed a good agreement with measured BF% in female wrestlers over 18 years of age, and no significant differences between both the measured and the predicted values, the Durnin and Womersley equation [19] predicted the most accurate BF% against measured BF% with the lowest bias. These results suggested that Durnin and Womersley [19], a prediction equation developed across a wide range of populations, provides the best estimate for female wrestlers. This may be because, although similar to Italian national female wrestlers [9], female wrestlers have a higher BF% compared to other female athletes. Another reason may be that most of the prediction equations were developed on male athletes whose body fat% was much lower than female athletes [33].

Although this study included the equation developed on [15] or verified on wrestlers [22], neither the NCAA Lohman method [22] nor the Thorland equation [15] developed for high school wrestlers provided accurate results to estimate BF%, with significant differences and proportional bias between BF% measured with BOD POD and the predictions. Differences in the results may be due to differences in the derivation sample’s age, body composition, and wrestling experience. Lohman et al. [22] stated that biological characteristics including age and sex-related biological characteristics seem to be the main factors limiting the regression equations. Although the Thorland equation was developed on wrestlers, the participants in the study consisted of high school wrestlers (16.5 ± 1.1 years) who were a younger population compared to this study [15]. Another factor creating this difference may be that both the Lohman and Thorland equations do not include sex as a variable. Most studies on wrestlers did not mention the sex of the participants [10,26,34,35]. However, sex is one of the main determinants of these equations, as evidenced by its inclusion as a variable in many skinfold prediction equations [18,19,20,21]. In this study, sex alone explained the 63.9% variance in BF% measured by BOD POD. The findings suggest that sex should also be considered in further studies.

This study evaluated wrestlers by sex and age group to estimate age- and gender-specific equations. The most accurate equations for estimating the BF% were the same across age groups for both sexes, although minor differences were presented. This may be due to similar physical characteristics such as body mass and skinfold sites used in the formulations. Body mass, abdomen and thigh skinfold regions used in the Stewart and Hannan equation and biceps, triceps, subscapular and iliac crest skinfold sites in the Durnin and Womersley equation did not differ statistically between age groups.

In this study, the results of multiple linear regression analysis proposed a specific prediction equation only using abdominal skinfold and sex as variables, explaining 83.2% of the variance to predict BF% measured by BOD POD. The most common prediction equations in wrestlers like Lohman [22] and Thorland [15] equations use triceps, subscapular and abdominal skinfold in the equations. Looking at the equations that make the most accurate BF% estimation in this study, the Stewart and Hannan equation [14] uses body mass, thigh, and abdominal skinfold sites to estimate BF%, while Durnin and Womersley [19] uses four skinfold sites, including the biceps, triceps, subscapular, and iliac crest skinfold. The use of the skinfold equation developed in the study may provide advantages in practical sports practice as it can reduce the time spent measuring other skinfold sites.

This study has some strengths. Both male and female wrestlers from different age groups were included to assess the effectiveness of gender and age. Twelve prediction equations were evaluated, including the three most common prediction equations in the literature, and nine equations developed on athletes, including specific equations for wrestlers. To eliminate measurement-related errors, Skinfold measurements were applied the same, well-experienced physiotherapist in line with the International Society for the Advancement of Kinanthropometry guidelines [28]. Several conditions were controlled before all measurements, including hydration status, dietary intake, exercise, the temperature and humidity of the measurement room. The study also has some limitations. At first, small sample size of female wrestlers (*n* = 25) needs to be addressed. In addition, using the BOD POD as a criterion method may create some limitations in comparing our results with the literature. Hydrostatic weighing was used as the criterion method in most studies on wrestlers [10,26,34,35]. However, the BOD POD technology is one of the gold standard methods validated in collegiate wrestlers against hydrostatic weighting [34,35]. In addition, Utter et al. [34] emphasized that the most important point to be considered when measuring body fat%, regardless of the method used, is to measure while the athlete is hydrated. Therefore, the body hydration status of the participants was carefully checked before the measurement process.

## 5. Conclusions

The equation developed by Stewart and Hannan for male wrestlers and the Durnin and Womersley equation for female wrestlers offered the best precision and lowest bias in BF% estimation compared to BOD POD. Apart from the good estimates of the Evans equations (3 SKF and 7 SKF) for female wrestlers over the age of 18, other equations underestimated or overestimated the BF% measured by BOD POD even they have been developed on athletic populations. These results suggest that caution should be taken when choosing a prediction equation for athletes as accuracy may be limited to the derivation sample. In addition, this study provided a new equation for estimating BF% in Olympic wrestlers using only the abdomen skinfold site and gender as variables. Requiring the measurement of only the abdomen skinfold site, this equation can provide a time-saving and practical approach for field measurements. Also, cross-validation of the equation developed may be considered to improve prediction accuracy of BF% in athletic populations.

## Figures and Tables

**Table 1:** Physical characteristics of the Olympic Wrestlers ^a^.

	Male Wrestlers (*n* = 61)		Female Wrestlers (*n* = 25)	
	<18 Age (*n* = 33)	≥18 Age (*n* = 28)	<18 Age (*n* = 14)	≥18 Age (*n* = 11)
Age (years)	16.24 ± 0.79	19.78 ± 1.57 *	16.21 ± 0.97	19.00 ± 1.09 *
Height (cm)	169.41 ± 7.61 ^¶^	171.33 ± 6.21 ^¶^	162.31 ± 7.51 ^¶^	159.93 ± 6.76 ^¶^
Body mass (kg)	70.45 ± 15.2 ^¶^	74.74 ± 10.88 ^¶^	60.95 ± 8.76 ^¶^	59.48 ± 11.69 ^¶^
BMI (kg.m^−2^)	24.35 ± 3.89	25.50 ± 2.86 ^¶^	23.11 ± 2.01	23.07 ± 3.17 ^¶^
**Skinfolds (mm)**				
Biceps	4.32 ± 1.47	4.10 ± 0.98	4.08 ± 0.86	4.56 ± 1.42
Triceps (mm)	8.97 ± 2.61	7.92 ± 2.70 ^¶^	10.71 ± 3.36	11.85 ± 4.52
Subscapular (mm)	10.31 ± 3.79	10.22 ± 2.74	8.62 ± 1.86	9.03 ± 2.65
Iliac crest (mm)	7.22 ± 4.01	6.31 ± 2.37	5.98 ± 1.68	7.12 ± 2.24
Abdominal (mm)	11.44 ± 6.57	10.19 ± 4.80	9.35 ± 3.11	11.23 ± 4.69
Midaxillary (mm)	7.84 ± 3.63	7.36 ± 2.89	6.48 ± 1.94	6.96 ± 2.34
Chest (mm)	6.55 ± 2.06 ^¶^	5.25 ± 1.50 *	5.11 ± 1.35 ^¶^	6.14 ± 1.16
Anterior thigh (mm)	11.53 ± 5.24 ^¶^	10.36 ± 4.52 ^¶^	17.34 ± 3.57 ^¶^	18.54 ± 6.93
Medial calf (mm)	7.39 ± 2.72 ^¶^	9.24 ± 4.04 *	9.88 ± 4.97 ^¶^	9.56 ± 4.55
**Sum of skinfolds (mm)**				
Sum of 2 skinfolds (mm) ^b^	21.76 ± 10.19	20.41 ± 7.24	17.98 ± 4.77	20.27 ± 6.83
Sum of 3 skinfolds-1 (mm) ^c^	27.13 ± 9.11	25.51 ± 7.32	25.82 ± 6.18	27.85 ± 8.35
Sum of 3 skinfolds-2 (mm) ^d^	30.73 ± 11.81	28.33 ± 9.26	28.70 ± 7.30	32.12 ± 10.94
Sum of 4 skinfolds-1 (mm) ^e^	38.58 ± 15.34	35.70 ± 11.92	35.18 ± 9.08	39.09 ± 12.65
Sum of 7 skinfolds (mm) ^f^	63.89 ± 25.47	57.62 ± 18.85	63.62 ± 14.21	70.90 ± 21.66
Sum of 8 skinfolds (mm) ^g^	68.22 ± 25.30	61.72 ± 18.81	67.71 ± 14.16	75.47 ± 21.77
**Body Fat (%)**				
BODPOD	8.84 ± 5.47 ^¶^	9.38 ± 7.43 ^¶^	18.23 ± 4.18 ^¶^	19.31 ± 5.43 ^¶^
Durnin and Womersley	15.62 ± 3.86 ^¶^	12.39 ± 2.72 * ^¶^	20.03 ± 2.73 ^¶^	19.69 ± 3.02 ^¶^
Jackson and Pollock (3SKF)	8.02 ± 3.56 ^¶^	7.33 ± 2.78 ^¶^	14.42 ± 2.67 ^¶^	15.67 ± 4.17 ^¶^
Jackson and Pollock (7SKF)	8.14 ± 3.42 ^¶^	7.69 ± 2.67	13.95 ± 2.27 ^¶^	15.21 ± 3.32
Lohman	11.31 ± 3.54	10.59 ± 2.79	10.71 ± 2.21	11.74 ± 3.28
Forsyth and Sinning (2SKF)	12.52 ± 5.60	11.79 ± 4.12	10.29 ± 2.67	11.52 ± 3.86
Forsyth and Sinning (4SKF)	12.21 ± 5.71	11.20 ± 4.20	10.78 ± 3.07	12.37 ± 4.93
Stewart and Hannan	8.61 ± 4.81	8.04 ± 3.98 ^¶^	11.16 ± 3.53	12.62 ± 5.46 ^¶^
Thorland et al. (3SKF)	10.55 ± 4.37	9.78 ± 3.61	9.96 ± 3.07	10.93 ± 4.03
Thorland et al. (3SKF)-2	11.67 ± 3.55	10.95 ± 2.80	11.06 ± 2.22	12.09 ± 3.29
White et al.	7.98 ± 2.56	7.38 ± 1.74 ^¶^	8.90 ± 1.30	9.58 ± 2.36 ^¶^
Evans et al. (3SKF)	10.53 ± 3.19 ^¶^	9.67 ± 2.69 ^¶^	18.22 ± 2.30 ^¶^	19.26 ± 3.80 ^¶^
Evans et al. (7SKF)	10.22 ± 3.07 ^¶^	9.46 ± 2.28 ^¶^	18.25 ± 1.71 ^¶^	19.13 ± 2.62 ^¶^

^a^ mean ± SD, range in parentheses. ^b^ subscapular, abdominal; ^c^ triceps, subscapular, midaxillary; ^d^ triceps, subscapular, abdominal ^e^ subscapular, abdominal, midaxillary, triceps; ^f^ Chest, Midaxillary, Triceps, Subscapular, Abdominal, Iliac crest, Thigh. ^g^ Chest, Midaxillary, Triceps, Subscapular, Abdominal, Iliac crest, Thigh, biceps. Abbreviations: BMI: Body mass index; SKF: Skinfold. * *p* < 0.05; analyzes between age groups of the same sex; ^¶^  *p* < 0.05; analyzes between age groups by sex.

**Table 2:** Coefficient of variation, absolute and relative technical error of measurement for anthropometric variables in Olympic wrestlers (*n* = 89).

Skinfolds	Coefficient of Variation (%)	Absolute (mm)	Relative (%)
Biceps	0.62	0.09	1.96
Triceps	0.62	0.07	0.73
Subscapular	0.55	0.08	0.79
Iliac crest	0.97	0.14	1.27
Abdominal	0.65	0.09	0.89
Midaxillary	0.77	1.11	1.01
Chest	0.82	0.09	1.57
Anterior Thigh	0.36	1.04	1.25
Medial Calf	1.70	0.14	1.56

**Table 3:** Correlations, bias, limits of agreement, and standardized differences between alterations in body fat percentage measured by BODPOD and other practical estimates in Olympic wrestlers.

	Male Olympic Wrestlers (*n* = 61)					
	<18 Age (*n* = 33)			≥18 Age (*n* = 28)		
Estimates of BF%	Correlation (95 CI%)	Bias (±LoA)	Standardized Differences (95% CI)	Correlation (95 CI%)	Bias (±LoA)	Standardized Differences (95% CI)
Durnin and Womersley	0.57 (0.26; 0.77)	−6.76 *(±7.20) ^a^	−1.8 (−2.38; −1.26)	0.65 (0.32; 0.84)	−4.23 * (±6.49) ^a^	−1.24 (−1.76; −0.76)
Jackson and Pollock (3SKF)	0.54 (0.19; 0.78)	0.82 (±6.70) ^a^	0.24 (−0.11; 0.58)	0.76 (0.53; 0.88)	0.82 (±5.09) ^a^	0.31 (−0.07; 0.68)
Jackson and Pollock (7SKF)	0.52 (0.15; 0.78)	0.70 (±6.87) ^a^	0.19 (−0.15; 0.54)	0.77 (0.55; 0.89)	0.46 (±5.27) ^a^	0.17 (−0.20; 0.54)
Lohman	0.49 (0.11; 0.74)	−2.47 * (±7.01) ^a^	−0.67 (−1.06; −0.30)	0.75 (0.50; 0.88)	−2.44 * (±5.20) ^a^	−0.89 (−1.34; −0.47)
Forsyth and Sinning (2SKF)	0.46 (0.09; 0.74)	−3.68 * (±7.35)	−0.96 (−1.38; −0.55)	0.68 (0.41; 0.85)	−3.63 * (±5.23)	−1.32 (−1.85; −0.83)
Forsyth and Sinning (4SKF)	0.48 (0.11; 0.75)	−3.36 * (±7.32)	−0.88 (−1.29; −0.48)	0.74 (0.47; 0.87)	−3.05 * (±5.11)	−1.14 (−1.63; −0.68)
Stewart and Hannan	0.52 (0.16; 0.76)	0.23 (±7.18)	0.06 (−0.28; 0.40)	0.78 (0.55; 0.89)	0.12 (±4.77)	0.05 (−0.32; 0.41)
Thorland et al. (3SKF)	0.50 (0.13; 0.75)	−1.71 * (±7.57)	−0.43 (−0.79; −0.08)	0.76 (0.51; 0.89)	−1.63 * (±5.53)	−0.56 (−0.96; −0.17)
Thorland et al. (3SKF)−2	0.49 (0.13; 0.75)	−2.83 * (±7.01) ^a^	−0.77 (−1.17; −0.39)	0.75 (0.49; 0.88)	−2.80 * (±5.20) ^a^	−1.02 (−1.50; −0.58)
White et al.	0.56 (0.19; 0.80)	0.87 (±7.43) ^a^	0.22 (−0.12; 0.57)	0.71 (0.44; 0.86)	0.77 (±6.25) ^a^	0.24 (−0.13; 0.61)
Evans et al. (3SKF)	0.54 (0.16; 0.79)	−1.69 * (±7.07)^a^	−0.46 (−0.82; −0.10)	0.80 (0.60; 0.89)	−1.52 * (±5.17) ^a^	−0.56 (−0.96; −0.17)
Evans et al. (7SKF)	0.53 (0.17; 0.79)	−1.38 * (±6.99)^a^	−0.38 (−0.73; −0.03)	0.78 (0.55; 0.90)	−1.31 * (±5.54) ^a^	−0.45 (−0.84; −0.07)
	**Female Olympic Wrestlers (*n* = 25)**					
	**<18 age (*n* = 15)**			**≥18 age (*n* = 10)**		
**Estimates of BF%**	**Correlation** **(95 CI%)**	**Bias (±LoA)**	**Standardized Differences** **(95% CI)**	**Correlation** **(95 CI%)**	**Bias (±LoA)**	**Standardized Differences** **(95% CI)**
Durnin and Womersley	0.62 (0.14; 0.87)	−1.80 (±6.41)	−0.52 (−1.08; 0.02)	0.81 (0.36; 0.95)	0.46 (±4.70)	0.18 (−0.42; 0.78)
Jackson and Pollock (3SKF)	0.65 (0.19; 0.88)	3.81 * (±6.20)	1.13 (0.48; 1.84)	0.58 (−0.17; 0.99)	4.34 * (±5.41)	1.44 (0.59; 2.41)
Jackson and Pollock (7SKF)	0.73 (0.33; 0.91)	1.28 * (±1.37) ^¥^	1.37 (0.66; 2.15)	0.81 (0.38; 0.95)	4.91 * (±4.61)	1.91 (0.91; 3.09)
Lohman	0.68 (0.24; 0.89)	1.69 * (±1.47) ^¥^	2.27 (1.32; 3.38)	0.42 (−0.45; 0.96)	8.41 * (±4.78)	3.15 (1.69; 4.93)
Forsyth and Sinning (2SKF)	0.68 (0.23; 0.89)	7.95 * (±6.04)	2.43 (1.42; 3.59)	0.54 (−0.25; 1.00)	8.61 * (±4.66)	3.31 (1.79; 5.17)
Forsyth and Sinning (4SKF)	0.68 (0.23; 0.89)	7.45 * (±6.03)	2.28 (1.32; 3.38)	0.42 (−0.46; 0.96)	7.65 * (±5.74)	2.39 (1.21; 3.79)
Stewart and Hannan	0.71 (0.36; 0.92)	7.07 * (±5.68)	2.3 (1.33; 3.41)	0.73 (0.19; 0.93)	7.18 * (±7.03)	1.83 (0.85; 2.97)
Thorland et al. (3SKF)	0.66 (0.20; 0.88)	8.27 * (±6.17)	2.47 (1.45; 3.65)	0.50 (−0.30; 0.92)	9.10 * (±5.10)	3.20 (1.72; 5.00)
Thorland et al. (3SKF)−2	0.68 (0.24; 0.89)	1.64 * (±1.47) ^¥^	2.17 (1.24; 3.23)	0.42 (−0.48; 0.96)	8.05 * (±4.78)	3.02 (1.61; 4.73)
White et al.	0.71 (0.29; 0.90)	9.33 * (±6.62) ^a^	2.6 (1.54; 3.83)	0.78 (0.30; 0.95)	2.09 * (±1.32) ^¥^	3.66 (2.00; 5.69)
Evans et al. (3SKF)	0.67 (0.22; 0.89)	0.01 (±6.16) ^a^	0.004 (−0.51; 0.52)	0.59 (−0.18; 1.00)	0.83 (±5.28)	0.28 (−0.32; 0.90)
Evans et al. (7SKF)	0.73 (0.32; 0.91)	−0.01 (±6.19) ^a^	−0.004 (−0.52; 0.51)	0.82 (0.39; 0.96)	1.07 (±4.76)	0.40 (−0.21; 1.04)

Significant differences between body fat percentage measured by BODPOD and other practical estimates using paired sample *t* test * (*p* < 0.05). ^¥^: Calculated with back transform of log-transformation. ^a^: There is proportional bias. Abbreviations: BF: Body fat; CI: Confidence Interval; LoA: level of agreement; SKF: Skinfold.

**Table 4:** Correlation between body fat percentage measured by BODPOD and skinfold measurements in Olympic wrestlers.

Variable	r	*p*
**Skinfolds**		
Biceps	−0.012	0.912
Triceps	0.514	0.001
Subscapular	0.282	0.009
Iliac crest	0.442	0.001
Abdominal	0.448	0.001
Midaxillary	0.359	0.001
Chest	0.258	0.017
Anterior thigh	0.684	0.001
Medial calf	0.290	0.007
**Sum of Skinfolds**		
Sum of 2 skinfolds (mm) ^a^	0.396	0.001
Sum of 3 skinfolds-1 (mm) ^b^	0.460	0.001
Sum of 3 skinfolds-2 (mm) ^c^	0.483	0.001
Sum of 4 skinfolds-1 (mm) ^d^	0.464	0.001
Sum of 7 skinfolds (mm) ^e^	0.551	0.001

^a^ subscapular, abdominal; ^b^ triceps, subscapular, midaxillary; ^c^ triceps, subscapular, abdominal; ^d^ subscapular, abdominal, midaxillary, triceps; ^e^ Chest, Midaxillary, Triceps, Subscapular, Abdominal, Iliac Chest, Thigh. Abbreviations: BMI: Body mass index; SKF: Skinfold.

**Table 5:** Developed models for body fat percentage prediction for Olympic wrestlers.

Model	R^2^	Adjusted R^2^	SEE	R^2^ Change	F	F Change	Significant F Change
1	0.639	0.635	3.943	0.639	133.1	133.1	<0.001
2	0.832	0.827	2.711	0.193	183.1	84.705	<0.001
3	0.838	0.831	2.682	0.006	125.6	2.5991	0.1112
4	0.844	0.835	2.647	0.006	97.42	2.929	0.0913
5	0.849	0.839	2.62	0.005	80.07	2.511	0.1175
6	0.852	0.84	2.613	0.003	67.28	1.351	0.249
7	0.852	0.837	2.631	0	56.92	0.0792	0.7792

Model 1: sex; Model 2: sex, abdominal; Model 3: sex, abdominal, thigh; Model 4: sex, abdominal, thigh, triceps; Model 5: sex, abdominal, thigh, triceps, iliac crest; Model 6: sex, abdominal, thigh, triceps, iliac crest, age; Model 7: sex, abdominal, thigh, triceps, iliac crest, age, subscapular; R^2^ = coefficient of determination; SEE = standard error of the estimate.

## Data Availability

The data presented in this study are available on request from the corresponding author. The data are not publicly available due to the Ministry of Youth and Sports Policy.
